# The Intraocular Pressure-Lowering Effect of Persimmon leaves (*Diospyros kaki*) in a Mouse Model of Glaucoma

**DOI:** 10.3390/ijms20215268

**Published:** 2019-10-23

**Authors:** Hong Ryul Ahn, Jae Wook Yang, Jee Young Kim, Chang Yong Lee, Tae-Jin Kim, Sang Hoon Jung

**Affiliations:** 1 Natural Products Research Center, Korea Institute of Science and Technology (KIST), Gangneung 25451, Korea; hrahn@kist.re.kr; 2 T2B Infrastructure Center for Ocular disease, Department of Ophthalmology, Inje University Busan Paik Hospital, Busan 47392, Korea; oculoplasty@gmail.com (J.W.Y.); thioridazine@naver.com (J.Y.K.); 3 Department of Food Science, Cornell University, Ithaca, NY 14850, USA; cyl1@cornell.edu; 4 Department of Biological Sciences, Pusan National University, Busan 46241, Korea

**Keywords:** persimmon leaves, ocular hypertension, intraocular pressure, soluble guanylate cyclase, glaucoma

## Abstract

The aim of this study was to evaluate the pharmacological efficacy of persimmon leaves in two glaucoma models, microbeads-induced ocular hypertension (OHT) and DBA/2 mouse. Thus, we demonstrated that Ethanol Extract of *Diospyros kaki* (EEDK) reduced elevated intraocular pressure (IOP) in both mouse models of glaucoma by measurements with a tonometer. In particular, we revealed that retinal ganglion cell loss and optic nerve damage caused by IOP elevation were markedly diminished as assessed by TUNEL assay, H&E staining, and fluorescent staining, while the expression of soluble guanylate cyclase (sGCα-1) increased, when EEDK was administered, as revealed by western blot. Moreover, the b-wave magnitude indicating functional scotopic vision was significantly improved in EEDK-administered DBA/2 mice during the 10-week follow-up study, as observed with electroretinography. Collectively, our results suggested that EEDK could be an effective therapeutic and IOP-lowering agent for preventing and treating retinal degenerative diseases such as glaucoma.

## 1. Introduction

Glaucoma is a progressive optic neuropathy characterized by the loss of retinal ganglion cells (RGCs) and their axons, which constitute the optic nerve [1]. The degeneration of RGCs causes damage to the visual field, and consequently results in complete blindness if left untreated [2]. Since this disease is multifactorial and strategies to cure it have not yet met the endpoints of clinical trials involving visual field recovery, treatment of the elevated intraocular pressure (IOP) remains the common practice. Thus, current therapies have been focused on reducing IOP in order to slow the glaucomatous progression [3].

The main pharmacological agents that are used for elevated IOP treatment include prostaglandin analogs, carbonic anhydrase inhibitors, and beta-blockers [4,5,6,7,8]. Laser trabeculoplasty and incision surgery are also alternative procedures to reduce IOP by regulating aqueous humor inflow and outflow pathways [9,10]. At present, topical prostaglandin analogs are the most widely used treatments to lower elevated IOP in patients with glaucoma and ocular hypertension, but could elicit several ocular side effects such as eyelash changes, conjunctival hyperaemia, and iris pigmentation [11,12]. If eye drops do not reduce the elevated IOP to the required level, carbonic anhydrase inhibitors can be used as oral medications, but still could pose side effects, including frequent urination, stomach upset, depression, and kidney stones [11,13]. Therefore, there is an increasing demand for the development of an effective IOP-lowering agent that is easier to administer, and safe, with minimum side effects even for long-term use.

Persimmon, *Diospyros kaki* Thunberg (Ebenaceae) is mostly grown in Eastern Asia, including Korea, China and Japan [14]. It is rich in lutein and zeaxanthin, both carotenoids that protect eyes from debilitating ocular diseases, such as cataracts and age-related macular degeneration (AMD) [15]. Like the fruit, the leaves contain abundant bioactive chemicals, including polyphenols, flavonoids, vitamins, and organic acids, most of which are known to exert beneficial pharmacological effects, such as strong radical-scavenging, antioxidant, and immune-modulatory properties [16,17,18]. Recent studies have shown that persimmon leaves exhibits antithrombotic potential by suppressing blood coagulation and platelet activation, as well as anticancer activity by inhibiting tumor growth [19,20]. Although the efficacy of persimmon leaves has been reported using several biological tissue samples, their role and underlying mechanisms in retinal tissue are underreported.

In our previous study, we showed that persimmon leaf extract has protective effects against retinal degeneration in mouse models of retinal degeneration [17,21]. However, it remains to be determined whether and how persimmon leaves could reduce in vivo elevated IOP. Thus, the aim of this study was to evaluate the pharmacological efficacy of persimmon leaves in two glaucoma models, microbeads-induced ocular hypertension (OHT) and DBA/2 mouse.

## 2. Results

### 2.1. IOP-Lowering Effects by EEDK in Microbeads-Induced OHT Mouse Model

**Figure 1:**
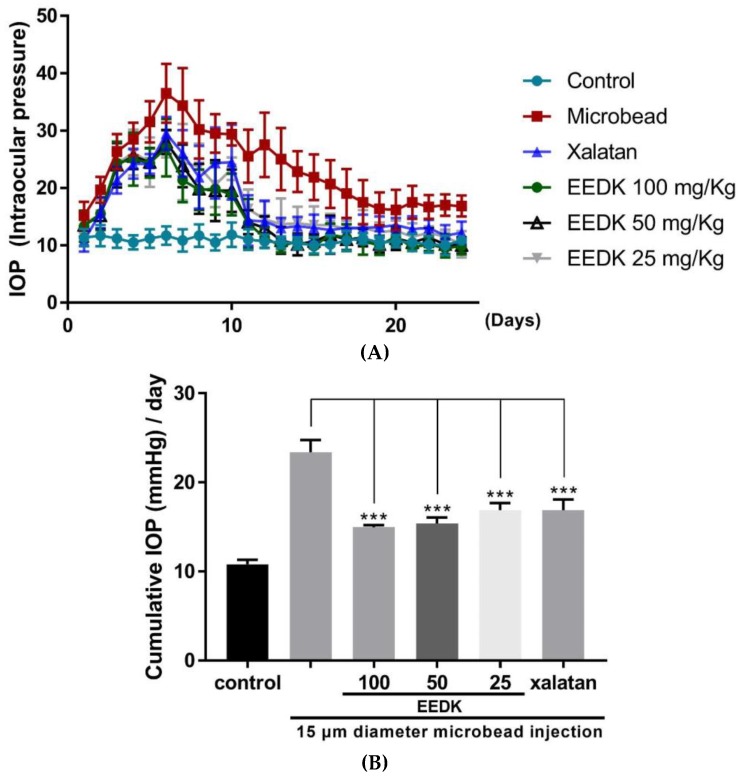
Effect of Ethanol Extract of *Diospyros kaki* (EEDK) on elevated intraocular pressure (IOP) in microbeads-induced ocular hypertension (OHT) mice. (**A**) Assessment of IOP elevation after microbeads injection. Values represent the mean ± S.E.M. for 6 animals. (**B**) Comparison of cumulative IOP value per day in experimental and control groups. Error bars represent standard error of the mean, *** *p <* 0.001.

Injecting microbeads into the anterior chamber of C57BL/6 mice provides a reliable methodology for developing an OHT-based glaucoma model. This mouse model is characterized by long-lasting IOP elevation with severe RGC death [22,23]. In this study, we examined whether EEDK could reduce the in vivo elevated IOP using this OHT mouse model. Immediately after injection of microbeads (2 µL), a large accumulation of beads was observed in the anterior segment in micrographs, which could easily block the Schlemm’s canal. The IOP-lowering effects of EEDK were compared with those of Xalatan, which is a medication currently used to treat glaucoma patients. As shown in Figure 1, IOP was measured every day until 24 days post injection for each experimental group. Maximum IOP levels were achieved at day 7 post injection in each group. The mean IOP peak at day 7 for each experimental condition was as follows: 10.83 ± 1.94 mmHg (*n* = 6) in control group, 34.33 ± 6.53 mmHg (*n* = 6) in the microbeads group, 26 ± 4.04 mmHg (*n* = 6) in Xalatan group, 24.5 ± 6.68 mmHg (*n* = 6) in the EEDK (25 mg/kg) group, 23.83 ± 3.71 mmHg (*n* = 6) in the EEDK (50 mg/kg) group, and 21.33 ± 3.88 mmHg (*n* = 6) in the EEDK (100 mg/kg) group.

As expected, the maximum IOP was evidenced in microbeads-induced group, and a relatively low value of IOP was measured in the group treated with EEDK or Xalatan. Notably, at 7 days post injection, IOP gradually decreased to normal baseline levels in all groups, but the decrease in the EEDK-treated group was much faster, eventually reaching levels similar to the control group. We further analyzed the cumulative IOP value per day, which could display more clearly the IOP lowering effect of EEDK. The numerical value measured was as follows: 10.83 ± 0.5 mmHg in control group, 23.39 ± 1.36 mmHg in the microbeads group, 14.97 ± 0.25 mmHg in the EEDK (100 mg/kg) group, 15.40 ± 0.67 mmHg in the EEDK (50 mg/kg) group, and 16.88 ± 0.81 mmHg in the EEDK (25 mg/kg) group, 16.88 ± 1.21 mmHg in Xalatan group (Figure 1B). These results demonstrated that oral administration of EEDK to the microbeads-induced OHT model induced a significant reduction of IOP with similar efficacy as that of topical application of Xalatan.

### 2.2. Protective Effect of EEDK on RGC Survival in OHT Model

**Figure 2:**
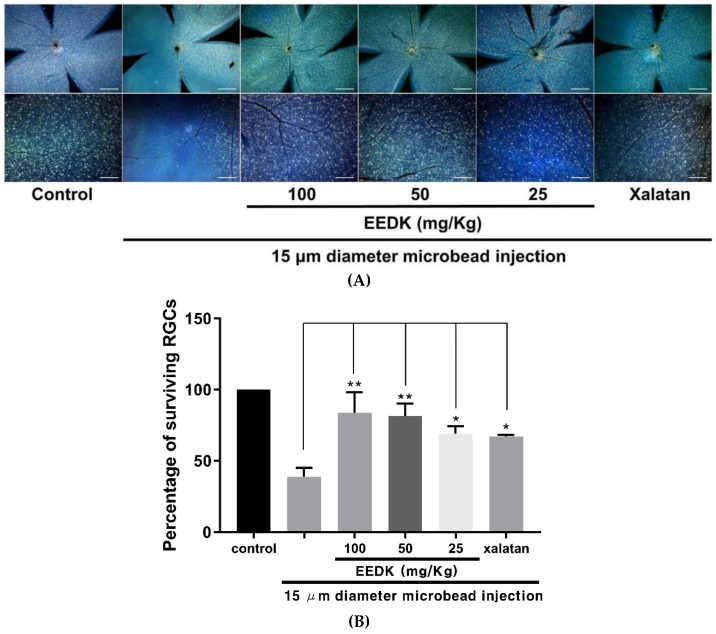
Effect of EEDK on RGCs survival in microbeads-induced OHT mice. (**A**) Representative fluorescent images of retrograde-labelled RGCs in microbeads-induced mice (Scale bars, upper panels: 500 μm, lower panels: 100 μm) (**B**) The bar graph shows quantitative analysis of the RGCs survival rate (%) compared with negative control. Values represent the mean ± S.E.M. for 3 animals (* *p* < 0.05, ** *p* < 0.01, mean ± S.E.M.).

Retrograde labelling of RGCs with fluorescent tracers has been widely used to determine RGC survival in a range of applications. This method provides a more accurate quantification of RGC survival because it excludes the interference of displaced amacrine cells located in the RGC layer [24,25]. Therefore, retinal cells were prepared together with Fluoro-Gold dye injected into the superior colliculus of the brain, and assessed 24 days after microbead injection. As shown in Figure 2A, retrograde-labelled RGCs in flat-mounted retinas of each group were imaged with fluorescent microscopic systems. Consistent with the IOP-lowering effects of EEDK, the survival rate of RGCs was significantly increased in EEDK-administered groups as follows: 83.72 ± 14.33%, 81.26 ± 8.83% in 100 mg/kg and 50 mg/kg EEDK groups, respectively, compared to that of the microbeads-induced group (38.78 ± 6.26%) (Figure 2B). These data suggested that oral administration of EEDK to the microbeads-induced OHT model prevented RGCs loss caused by ocular hypertension.

### 2.3. Effect of EEDK on the Expression of Soluble Guanylate Cyclase α-1 (sGCα-1)

**Figure 3:**
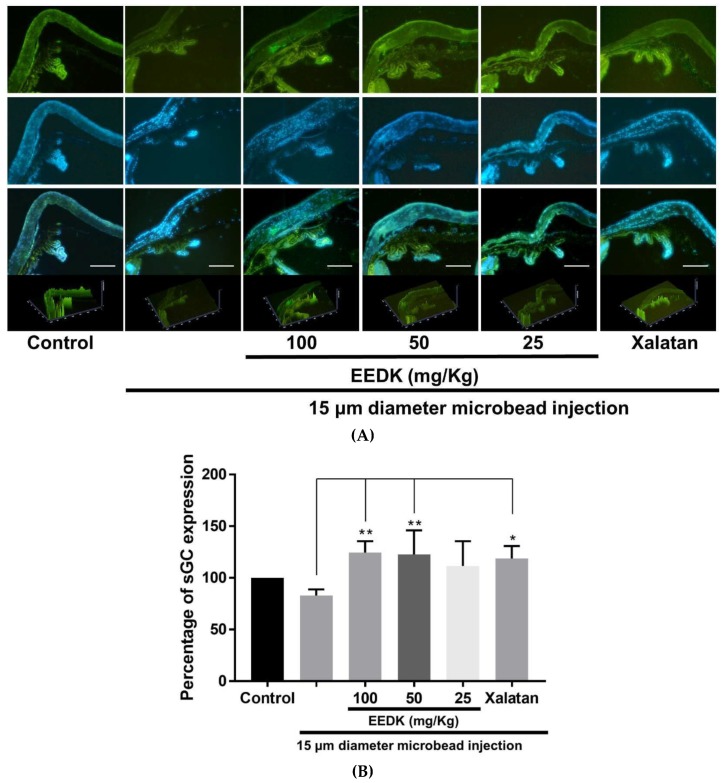
Effect of EEDK on sGCα-1 expression in microbeads-induced OHT mice. (**A**) Immunohistochemistry of the sGCα-1 protein expression in microbeads-induced mouse eye tissue. Expression of sGCα-1 protein and counterstaining (DAPI) are shown in green and blue colors, respectively. Scale bar = 100 μm. (**B**) The bar graph shows quantitative analysis of the sGCα-1 expression compared with negative control. Values represent the mean ± S.E.M. for 3 animals (* *p* < 0.05, ** *p* < 0.01, mean ± S.E.M.).

Since the soluble guanylate cyclase α-1 (sGCα-1) protein is a primary regulator for vascular hypertension, we hypothesized that EEDK could affect ocular hypertension by regulating sGCα-1 signals [26]. Therefore, we examined the expression level of sGCα-1 in each group to understand the mechanism by which EEDK regulates ocular hypertension. As shown in Figure 3A, the expression of sGCα-1 in EEDK-administered group was significantly increased in the angle part of the eyeball, ciliary body, compared to microbeads-induced ones without EEDK (green color: sGCα-1, blue color: counterstain, DAPI). The quantitative analysis of sGCα-1 expression in each group is shown in Figure 3B. In EEDK-administered groups (50 mg/kg and 100 mg/kg doses), the expression levels of sGCα-1 were significantly higher compared to the control, but similar to those obtained with Xalatan. These results suggested that EEDK could preserve the endogenous sGCα-1 expression in the anterior segment of OHT mouse model.

### 2.4. IOP Lowering Effects by EEDK in DBA/2 Glaucoma Model

**Figure 4:**
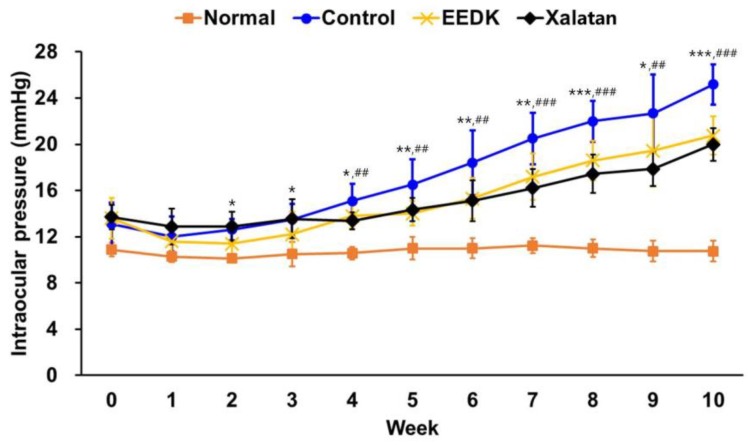
Comparison of intraocular pressure (IOP) of the DBA/2 mouse. IOPs for all groups were measured by a rebound tonometer. Values represent the mean ± S.E.M. for 10 animals. The values of * *p* < 0.05, ** *p* < 0.01 and *** *p* < 0.001 indicate significant difference when the EEDK-administered group was compared with control group. ^##^ *p* < 0.01 and ^###^ *p* < 0.001 indicate significant difference between the Xalatan-administered group and control group. IOPs in EEDK- or Xalatan-administered group were measured by a rebound tonometer.

In addition to the IOP-lowering effects of EEDK in OHT mouse model, we further examined whether EEDK had similar pharmacological efficacy in other types of glaucoma models. DBA/2 mice have been known as an inherited and age-related progressive glaucoma model [27]. The intraocular pressure of DBA/2 mice was measured once a week during the 10-week follow-up study from the adaptation period. As shown in Figure 4, normal mice (C57BL/6) exhibited no significant increase in IOP for 10 weeks, whereas IOP in DBA/2 mice continuously increased from 3 weeks, probably due to genetic and age-dependent glaucomatous progression [28]. Notably, when EEDK was administered, DBA/2 mice exhibited a lower level of IOP, similar to the pharmacological efficacy of Xalatan. Therefore, our results suggested that EEDK induced similar IOP-lowering effects in age-dependent glaucoma model as those in the microbeads-induced OHT model.

### 2.5. Effect of EEDK on ERG B-Wave Amplitude in DBA/2

**Figure 5:**
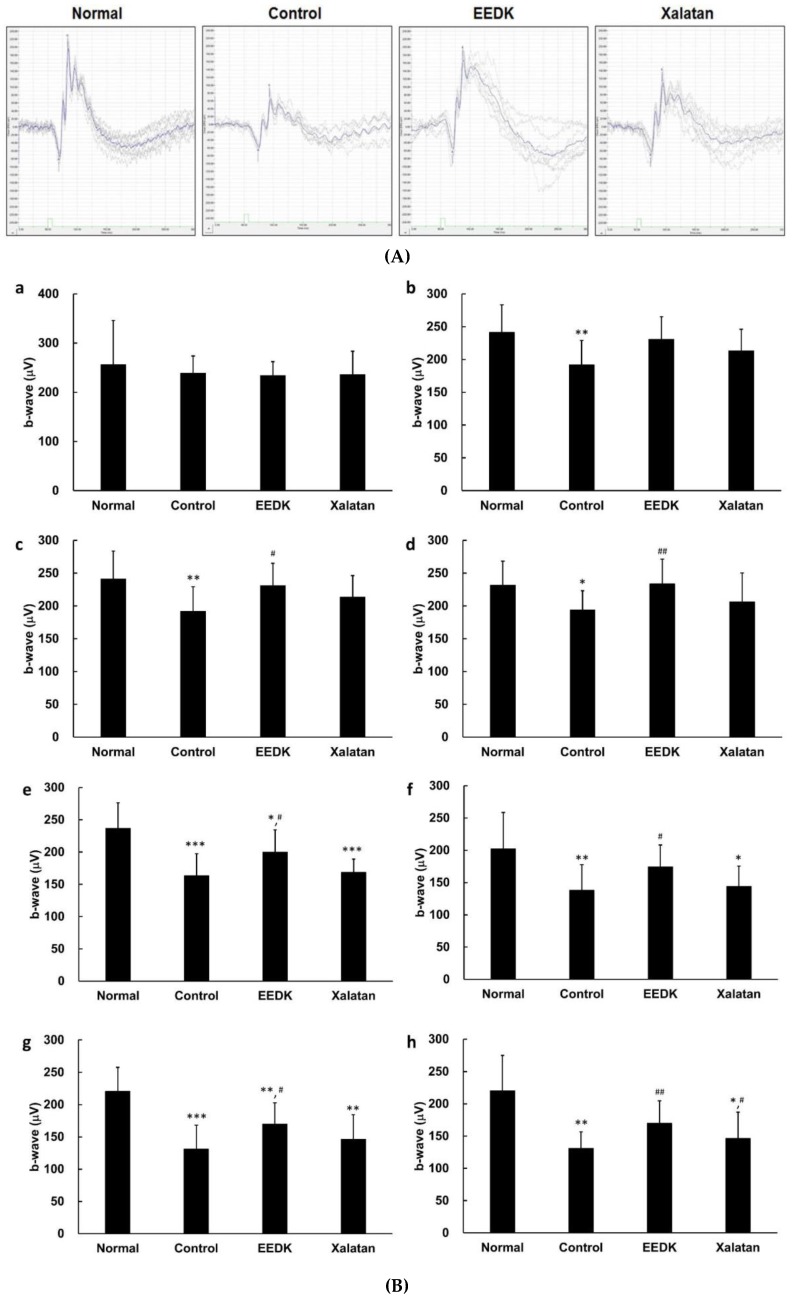
Effect of EEDK on b-wave amplitude in DBA/2 mouse. (**A**) Representative scotopic ERG graph. (**B**) B-wave amplitudes in scotopic conditions were analyzed by ERG at week 0 (a), week 2 (b), week 4 (c), week 6 (d), week 7 (e), week 8 (f), week 9 (g), and week 10 (h). Values represent the mean ± S.E.M. for 10 animals. The values of * *p* < 0.05, ** *p* < 0.01 and *** *p* < 0.001 indicate significant difference when comparing with normal group; meanwhile, ^#^ *p* < 0.05 and ^##^ *p* < 0.01 indicate significant difference of EEDK or Xalatan-administered group compared with control group.

Electroretinogram (ERG) is extensively used in eye research for the diagnosis of various retinal disorders, including glaucoma, because this tool can provide information about retinal degeneration by measuring the electrical response of the light-sensitive retinal cells [29]. We used this system to compare the ERG recording for each group and to periodically monitor the in vivo efficacy of EEDK. Figure 5A shows the representative scotopic ERG graph acquired from this study, and the values of b-wave amplitude in scotopic conditions were compared in each group during the 10-week follow-up study. Although there was no significant difference in the amplitude of b-wave between groups immediately after the refinement period of the experimental animals (Figure 5B-a), the b-wave values of the control group significantly decreased from the 2 weeks after starting the test, as shown in Figure 5B-(b–h). The efficacy for EEDK administration was confirmed 4 weeks after beginning of the experiment, according to Figure 5B-(c–h). After 4 weeks, the amplitude of b-wave in EEDK-treated group considerably increased compared with that of the control group (DBA/2). When Xalatan was used as a counter substance, the b-wave almost reached similar levels to the ones obtained with the control 7 weeks after the initiation of the test, but increased in the last 10 weeks compared to the control mice. Therefore, these results suggested that EEDK could improve the b-wave response in glaucoma mouse model.

### 2.6. Effect of EEDK on Optic Nerve Head Damage in DBA/2 Mouse

**Figure 6:**
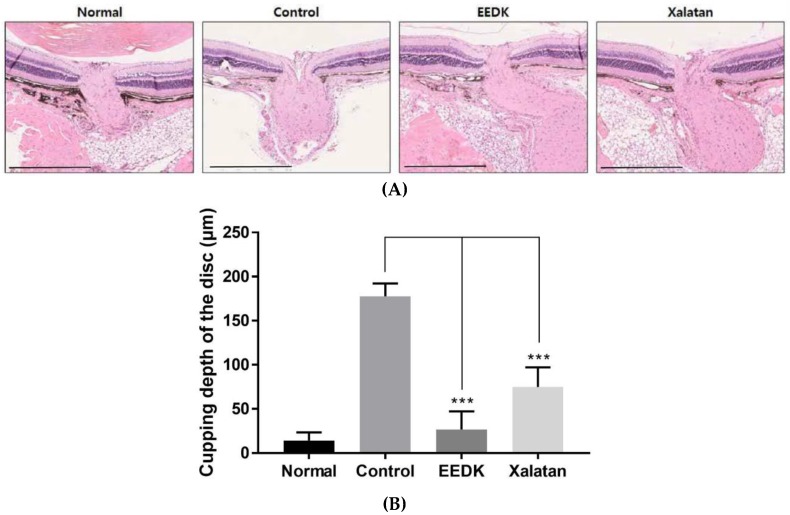

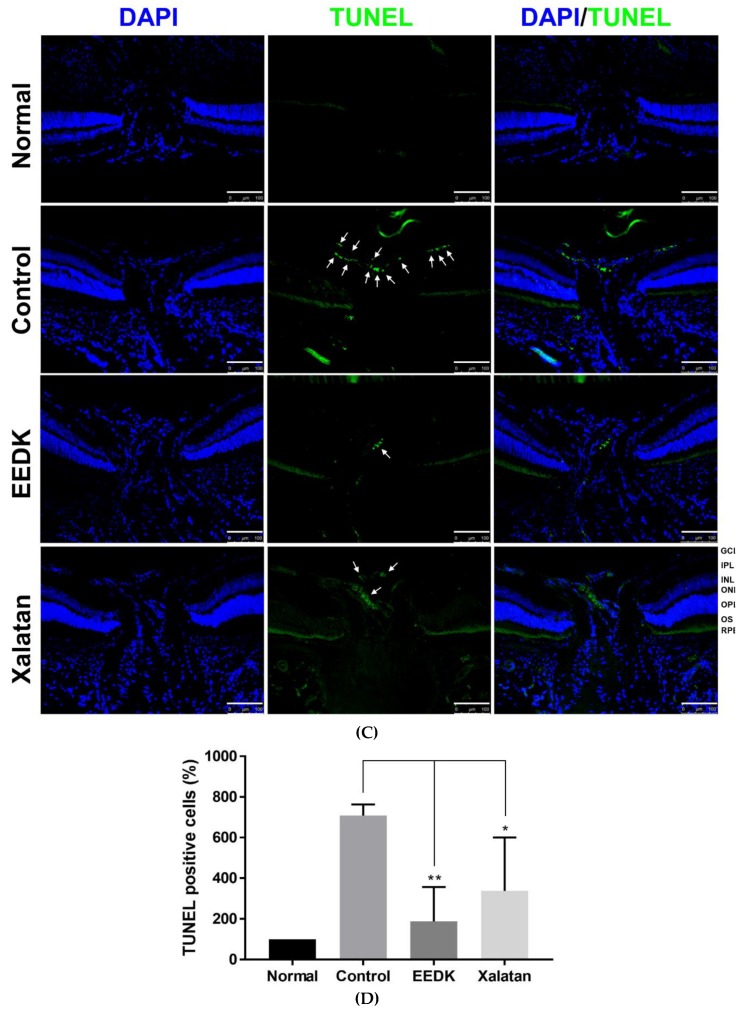
Effect of EEDK on optic nerve head damage in DBA/2 mouse. (**A**) Enucleated eyeballs were fixed for paraffin embedding. The tissue slides were stained by H&E solution. Optic nerve heads of retinas were observed using Nanozoomer. Scale bar = 500 μm (**B**) The bar graph shows quantitative analysis of the optic disc cupping. Values represent the mean ± S.E.M. for 3 animals (*** *p* < 0.001, mean ± S.E.M.). (**C**) Cell death in damaged tissue was analyzed by TUNEL assay. The tissue on glass slide was fixed and immunostained with TUNEL assay kit, according to the manufacturer’s protocol. The immunoreactivity was observed by fluorescence microscope. Scale bar represents 100 μm. GCL, ganglion cell layer; IPL, inner plexiform layer; INL, inner nuclear layer; OPL, outer plexiform layer; ONL, outer nuclear layer; OS, outer segment; RPE, retinal pigment epithelia; ON, optic nerve. (**D**) The bar graph shows quantitative analysis of the TUNEL positive cell rate (%). Values represent the mean ± S.E.M. for 3 animals (* *p* < 0.05, ** *p* < 0.01, mean ± S.E.M.).

The optic nerve is composed of numerous nerve fibers located at the back of the eye that carry visual information from the retina to the brain [30]. Although one major risk factor resulting in glaucomatous progression is eye pressure, different factors including genetic mutations have also been implicated in this process, causing serious optic nerve damage [31]. It is crucial to determine whether EEDK is also involved in optic nerve protection in DBA/2 mice. Since the degree of optic disc cupping is related to the size of the optic disc, and cup depth is a useful measure that reflects the anatomical changes in glaucoma, we compared the damage area of the optic nerve in each group with H&E staining. As shown in Figure 6A,B, the strongest protection of optic nerve head occurred in DBA/2 mice when EEDK was administered. Xalatan also reduced the optic nerve damage compared to control group. These results were further confirmed by the TUNEL assay, which is the common method to detect cellular death. The TUNEL-positive cells (green) were intensively detected in the optic nerve head of the control mice (DBA/2), while they were obviously diminished in the optic nerve head of the EEDK-administered group, indicating less apoptosis, a result similar to the one obtained in the Xalatan-treated group (Figure 6C,D). These data suggested that EEDK played a protective role in the optic nerve of DBA/2 mice.

### 2.7. Reduced RGCs Loss in EEDK-administered DBA/2 Mice

**Figure 7:**
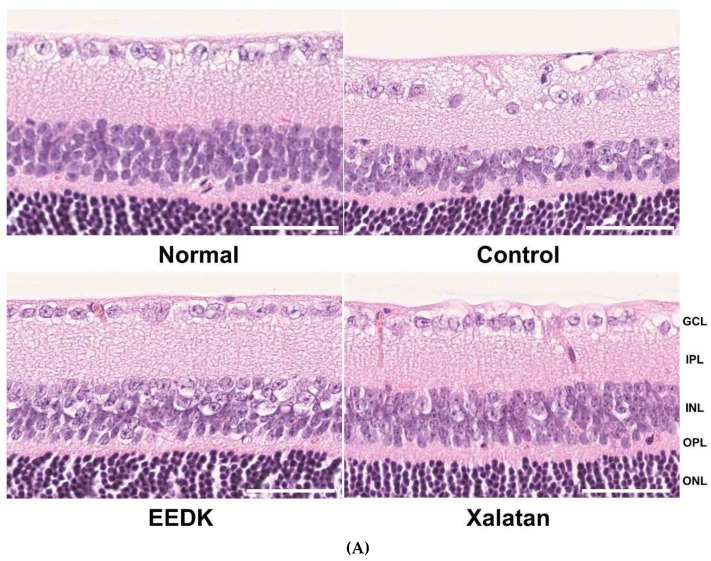

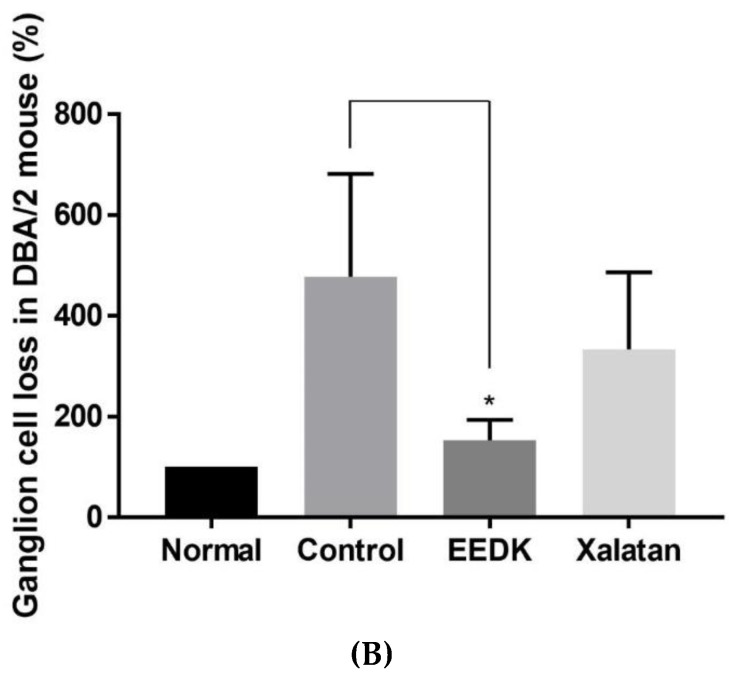
Effect of EEDK on ganglion cell loss in DBA/2 mouse. (**A**) Enucleated eyeballs were fixed for paraffin embedding. The tissue slides were stained with H&E solution, and observed using Nanozoomer. The scale bar represents 50 μm. GCL, ganglion cell layer; IPL, inner plexiform layer; INL, inner nuclear layer; OPL, outer plexiform layer; ONL, outer nuclear layer. (**B**) The bar graph shows quantitative analysis of the ganglion cell loss rate (%). Values represent the mean ± S.E.M. for 3 animals (* *p* < 0.05, mean ± S.E.M.).

We further examined whether EEDK could prevent RGC loss from glaucomatous defects in DBA/2 mice in each group of the retinas. Figure 7 shows representative photomicrographs exhibiting the histological appearance of retinal cross-sections following H&E staining. In control group (DBA/2), the number of ganglion cells decreased in comparison with the normal mice (C57BL/6), whereas the EEDK administration prevented retinal cells from RGCs degeneration in DBA/2 mice. Xalatan administration had a similar effect. In Xalatan-treated group, RGC loss was clearly abated, but partially presented in some areas, suggesting that EEDK played a role in RGC survival against RGC degeneration in DBA/2.

### 2.8. Effect of EEDK on Apoptotic Protein Expression in DBA/2 Mice

**Figure 8:**
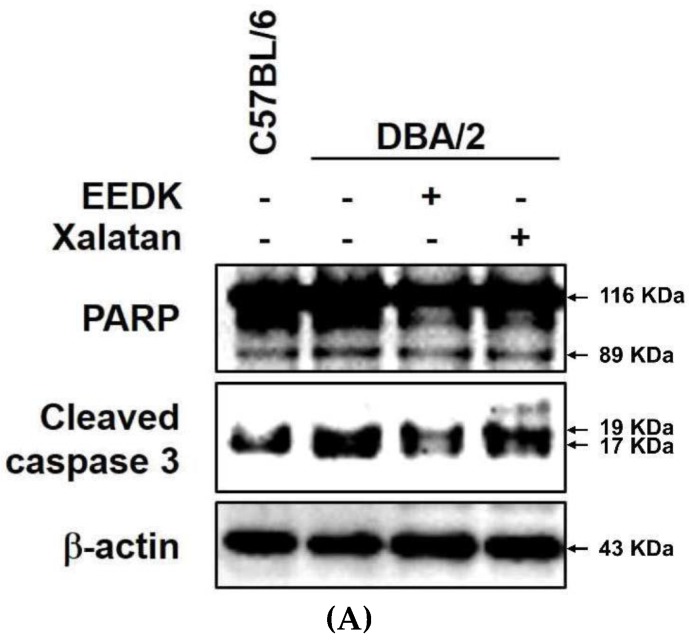

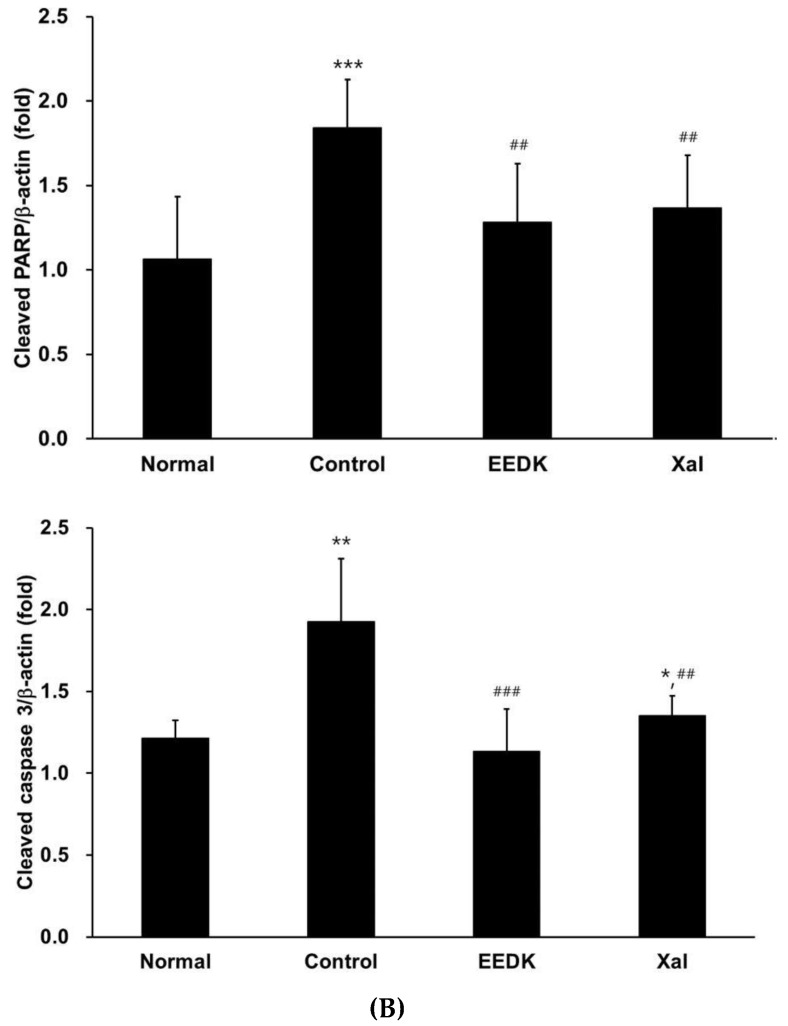
Effect of EEDK on apoptotic protein expression in DBA/2 mouse. (**A**) Expression of apoptotic proteins: PARP and cleaved caspase-3, and β-actin (control) in DBA/2 mouse retinas by western blot. (**B**) Densitometries of cleaved PARP and cleaved caspase 3 protein bands were measured with Image J. Values represent the mean ± S.E.M. of 6–8 animals. Values of * *p* < 0.05, ** *p* < 0.01 and *** *p* < 0.001 indicate significant difference between each group compared with normal group. Meanwhile, ^##^ *p* < 0.01 and ^###^ *p* < 0.001 correspond to difference between the EEDK- or Xalatan-administered group with control group.

The expression of Poly (ADP-ribose) polymerase (PARP) and cleaved caspase 3 proteins associated with apoptosis was assessed in each group, as the optic nerve damage and RGC death were detected in DBA/2 mouse retinas. As shown in Figure 8, the expression of both PARP and cleaved caspase 3 proteins was markedly higher in the retinas of control mice. However, EEDK (50 mg/kg)-administered mice presented significantly attenuated levels, compared with the control group. Even though the expression of two cell damage markers was significantly reduced in both EEDK- and Xalatan-treated group, no significant difference was observed between these two groups (* *p* < 0.05, ** *p* < 0.01, and *** *p* < 0.001, ^##^ *p* < 0.01, and ^###^ *p* < 0.001, *n* = 6–8). These results demonstrated that EEDK promoted RGC protection by regulating apoptotic proteins.

## 3. Discussion

Since IOP is an important risk factor for glaucoma, agents that lower IOP have been considered reliable therapeutic approaches in the treatment of glaucoma [32]. However, the generation of an optimal animal model which develops the typical regressive pattern of glaucoma is challenging [33]. In this study, we effectively created a microbeads-induced ocular hypertension glaucoma model, and compared the results with those of DBA/2 mice, as both animal models develop age-dependent glaucoma phenotype. Additionally, both animal models are able to effectively raise and sustain IOP, which is optimal for validating the pharmacological efficacy of the EEDK. Indeed, IOP in both DBA/2 and microbeads-induced OHT mouse models was elevated to a level significantly higher than that of control mice (C57BL/6); meanwhile, EEDK administration resulted in values similar to those observe with Xalatan, which is a common medication used for patients diagnosed with glaucoma.

Prostaglandin analogs have been widely used as IOP-lowering eye drops for more than a decade, and are considered first-line treatments for glaucoma [34]. Mainly eye drops are used to protect eyes from glaucoma, but oral agents are still rare. Recently, the carbonic anhydrase inhibitors (CAIs), such as acetazolamide and neptzana, were approved by FDA as oral medications for glaucoma treatment. Although the oral administration is far more effective than the topical treatment, more systemic side effects, such as transient myopia, frequent urination, and paresthesia may occur during long-term use [35]. Therefore, it is very important to develop new oral agent candidates that have minimal side effects and toxicity, and are easy to ingest. Edible natural products can possibly fulfil such a role.

In our study, EEDK effectively treated glaucoma in the in vivo models. As EEDK contains several flavonoids, such as catechin, kaempferol, and quercetin, which are strong antioxidants and radical-scavengers, the beneficial effects of the extract on RGCs protection could be due to a synergistic effect between several of them [16], compared to the action of a single compound. The antioxidant properties of EEDK are important because the retina can easily become vulnerable to chronic oxidative stress, mainly triggered by high oxygen consumption, oxidization of polyunsaturated fatty acids, and excessive light exposure [36]. These unwanted, but inevitable factors are driven by retinal physiology, and as a consequence, accompanied by damage and apoptosis of retinal cells. Persimmon leaves also contain other beneficial compounds, including vitamin C, fiber and tannins, which support protection of RGCs [16,18]. In addition, certain bioactive components of EEDK would exert IOP-lowering effect via direct regulation of ciliary body. The ciliary body, containing the ciliary muscle, vessels, and fibrous connective tissues, produces the aqueous humor and controls its flow [37]. It is well known that aqueous humor must flow from the ciliary body into the anterior chamber, and out through the trabecular meshwork into a drainage canal [38]. If fluid does not flow properly through this route, it causes an increase in IOP, leading to optic neuropathy and vision loss [2,37]. The functional expression of sGCα-1 in ciliary body supported the idea that EEDK could regulate IOP in this context. Previous studies reported that optic neuropathy, involving retinal nerve fiber layer (RNFL) thinning and RGC loss, were highly associated with sGCα-1 protein expression because the deficiency of sGCα-1 in mice led to an increase of IOP and retinal vascular dysfunction [39]. Either abnormal or insufficient sGCα-1 expression in sGCα-1 deficiency mice can impair nitric oxide (NO)-cGMP signaling cascade, which consequently affects retinal blood pressure, attributing it to the glaucomatous phenotype. In our study, we firstly observed that EEDK-administered mice showed significant preservation of sGCα-1 compared with control group, suggesting that EEDK lowered IOP by regulating the functional expression of sGCα-1 in the ciliary body. EEDK may also impact on the myosin light chain (MLC) phosphorylation by inhibiting Rho kinase, since sGCα-1 is an upstream regulator of these two signals. Rho kinase and MLC activity play important roles in ciliary muscle contractility, which supports aqueous humor drainage. Thus, EEDK is likely to be involved in IOP regulation through sGCα-1 pathway.

In summary, we analyzed the pharmacological efficacy of EEDK in two animal models of glaucoma: microbeads-induced OHT and inherited, age-dependent glaucoma mouse models. In particular, we provided new evidence that EEDK could potentially lower IOP with notable RGCs/optic nerve protection against retinal degeneration. Furthermore, recordings from ERG confirmed that EEDK could play a protective role in scotopic vision in glaucoma mouse model. Taken together, our data suggested that EEDK may potentially be an effective therapeutic agent for preventing and treating retinal degenerative diseases such as glaucoma.

## 4. Materials and methods

### 4.1. Materials

The ethanol extract of persimmon leaves, *D. kaki* were extracted with ethanol in an extraction reactor and filtered through filter paper. The combined filtrate was concentrated and dried to obtain EEDK (yield: 11–17%). Lantanoprost (Xalatan eye drops) was purchased from Pfizer (New York, NY, USA).

### 4.2. Animals

All animal studies were performed in compliance with the ARVO Statement for the Use of Animals in Ophthalmic and Vision Research, and were approved by the Animal Care and Use Committee of two joint research institutions (KIST No. 2014-011, approval date: 23-06-2014, Inje University Busan Paik hospital IJUBPH-2016-008-01, approval date: 01-08-2016). Male C57BL/6 mice weighing 20–30 g (15 weeks of age) and 20–25 g (6 weeks of age) were used to evaluate the IOP-lowering and pharmacological efficacies of EEDK. DBA/2 mice weighing between 20–30 g (15 weeks of age) were also used for the study. The mice were adapted for a week after arrival, grouped into 6–10 mice, and had access to animal chow with water ad libitum. All mice were housed at temperature-controlled room (25 ± 1 °C) and 50% humidity, with a 12-h light-dark cycle prior to the beginning of the experiments.

### 4.3. Induction of Elevated IOP

Mice were anesthetized by intraperitoneal injection of a mixture of Zoletil (1.6 μg/g; Virbac Laboratories 06515, France) and Rompun (0.05 μL/g, Bayer plc, UK), supplemented with topical application of proparacaine (0.5%; Bausch &Lomb, Tampa, FL, USA). The IOP increase was induced by injecting of polystyrene-microbeads (FluoSpheres; Invitrogen, Carlsbad, CA, USA; 15 μm diameter) into the anterior chamber of each animal’s right eye under a surgical microscope. The cornea on the right was gently drilled around the center using a sharp glass micropipette (size: 100 ± 20 μm). A small volume (2 μL of 5.0 × 10^6^ beads/mL in phosphate-buffered saline (PBS)) of microbeads was injected through the hole of the anterior chamber, and then injection of an air bubble was performed via the micropipette connected with a Hamilton syringe. The same volume of PBS was injected in the control experiment on microbeads. Mice were placed on a heating pad set at warm temperatures for quick recovery after injection, and antibiotic ointment (Dechra Veterinary Products, Overland Park, KS, USA) was treated topically in the eyes to prevent infection [40].

### 4.4. IOP Measurement

IOP was measured with a TonoLab rebound tonometer (Colonial Medical Supply, Franconia, NH, USA), which automatically generated the mean of six measurements from each eye of ten mice, after elimination of the highest and lowest values; and the mean was used to calculate the mean IOP.

### 4.5. Western Blot Analysis

Retinal tissues were surgically dissected from both C57BL/6 and DBA/2 mice and washed in cold PBS. Tissues were homogenized in RIPA buffer (150 mM NaCl, 1.0% IGEPAL CA-630, 0.5% sodium deoxycholate, 0.1% sodium dodecyl sulphate [SDS], 50 mM Tris, pH 8.0, 1× protease inhibitors, and 1 mM PMSF) and centrifuged at 14,000× *g* for 30 min at 4 °C.

Total protein concentrations were determined by a Bio-Rad Protein Assay Kit (Bio-Rad, Hercules, CA, USA). Proteins (10 μg/lane) were loaded into a 10% SDS-polyacrylamide gel, subjected to electrophoresis and transferred to a PVDF (polyvinylidene difluoride) membrane (Amersham Biosciences, GE Healthcare, UK).

The following primary antibodies (diluted 1:1000): anti-β-actin (Santa Cruz, CA, USA), anti-poly(ADP-ribose) polymerase (PARP), and anti-cleaved caspase-3 (Cell Signaling Technology, MA, USA) were used for the membranes, which were washed with PBST (8 g/L NaCl, 0.2 g/L KCl, 1.44 g/L, Na_2_HPO_4_, 0.24 g/L, NaH_2_HPO_4_, and 0.1% Tween 20) and incubated with secondary antibodies (1:5000) at room temperature for 2 h. Protein bands were quantified by densitometry using ECL reagents (Amsersham Bioscience, UK), the Fusion FX5 system (Vilber Lourmat, Torcy, France), and ImageJ (v1.48, NIH, Bethesda, MD, USA).

### 4.6. Histological Analysis

Enucleated eyes were fixed in 10% formalin for 24 h, embedded in paraffin, and sectioned through the equatorial plane at a 5-μm thickness using a microtome (Leica Biosystems, Nussloch, Germany). Briefly, hematoxylin solution (0.1% hematoxylin plus 10% ammonium) was added to the retinal section for 3–5 min. The sections were then washed 3 times with distilled water, rinsed with 95% alcohol, and stained with 1% Eosin Y solution for 1 min. Eosin Y was washed off with an ethanol series of solutions (85, 90 and 100%), carboxylene, and xylene for 3 min each, and the sections were cover slipped with a mounting medium and scanned using a digital slide scanner (NanoZoomer 2.0 RS, Hamamatsu, Japan).

### 4.7. RGCs Labeling and Retinal Flat Mount Preparation

The detailed experiment is well explained in our previous study [17]. Briefly, the neurotracer dye, Fluoro-gold (5% solution in saline, Invitrogen, NY, USA) was injected to the superior colliculus of mice using a piece of soaked Gelfoam. The eyes were taken out seven days after the Fluoro-gold application, and the retinas were detached at the ora serrata, followed by cutting with a trephine near the optic nerve head. After four radial incisions, the retinal flat mounts were prepared on silane-coated microscope slides.

### 4.8. Electroretinography

The recordings for electroretinogram (ERG) were obtained utilizing Micron Ganzfeld ERG (Phoenix Research Labs, Pleasanton, CA, USA). DBA/2 mice were adapted to the dark for at least 12 h overnight prior to the experiment and rod cell response was assessed by scotopic testing. Pupils were dilated with topical Tropherine ophthalmic solution (Hanmi, Seoul, Korea). Once the pupil was fully inflated, hypromellose 2% (Samil, Seoul, Korea) was applied and the electrode was inserted. The ERG was recorded according to the manufacturer protocol.

### 4.9. Immunohistochemistry

Enucleated eyes were fixed in 10% formalin for 24 h, embedded in paraffin, and sectioned through the equatorial plane at a 4-μm thickness using a HM340E microtome (Walldorf, Germany). Briefly, sections were post-fixed for 5 min in 70% ethanol, and incubated with blocking solution for 90 min in 5% Bovine serum in 0.1 M PBST. Sections were incubated overnight at room temperature with primary antibody (anti guanylate cyclase alpha 1: Abcam, UK) in blocking solution. The next day, sections were washed for 3 times in 0.1 M PBST and incubated with secondary antibody (Alexa 488 goat anti-rabbit: Abcam, UK) and counterstain solution (DAPI: Sigma, USA) for 1 h. Sections were washed 3 times in 0.1 M PBST solution. The sections were analyzed with a fluorescence microscope (TE2000-U; Nikon, Japan).

### 4.10. Statistical Analysis

The data were expressed as the mean value ± the standard error of the mean (SEM). Statistical comparisons were conducted using one-way ANOVA, followed by Dunnett’s test. Statistical analyses were performed using GraphPad Prism, version 6.0 (GraphPad, San Diego, CA, USA). P < 0.05 was considered statistically significant.
